# Effects of Word Length on Eye Guidance Differ for Young and Older Chinese Readers

**DOI:** 10.1037/pag0000258

**Published:** 2018-06

**Authors:** Sha Li, Lin Li, Jingxin Wang, Victoria A. McGowan, Kevin B. Paterson

**Affiliations:** 1 Academy of Psychology and Behavior, Tianjin Normal University; 2 Department of Neuroscience, Psychology and Behaviour, University of Leicester

**Keywords:** eye movements during reading, Chinese, word length, eye guidance

## Abstract

Effects of word length on where and for how long readers fixate within text are preserved in older age for alphabetic languages like English that use spaces to demarcate word boundaries. However, word length effects for older readers of naturally unspaced, character-based languages like Chinese are unknown. Accordingly, we examined age differences in eye movements for short (2-character) and long (4-character) words during Chinese reading. Word length effects on eye-fixation times were greater for older than younger adults. We suggest this age difference is due to older adults’ saccades landing more rarely at optimal intraword locations, especially in longer words.

During reading, the eyes move along lines of text in a series of rapid movements (saccades) separated by brief pauses (fixations). In alphabetic languages, these eye movements are strongly influenced by characteristics of both the fixated word and the next word along (Rayner, 2009). Word length, in particular, has a major influence on where readers look and for how long. Specifically, longer words are more likely to be fixated (and so not skipped) and to receive longer fixations than short words (Brysbaert, Drieghe, & Vitu, 2005; Joseph, Liversedge, Blythe, White, & Rayner, 2009; Paterson, Almabruk, McGowan, White, & Jordan, 2015; Paterson, McGowan, & Jordan, 2013; Rayner, 1979; Rayner & McConkie, 1976; Rayner, Sereno, & Raney, 1996). Moreover, readers use parafoveal cues to word length to target their forward-moving saccades. These saccades tend to land within a location between the beginning and middle of words, which Rayner (1979) termed the *preferred viewing location* (PVL). However, saccades can land closer to the beginning letters of long than short words due to oculomotor error (Joseph et al., 2009; McConkie, Kerr, Reddix, & Zola, 1988; Paterson et al., 2013, 2015). Word length effects therefore provide an effective diagnostic of the efficiency of eye guidance during reading.

An important concern is whether these effects change with age. It is well-established in alphabetic languages that older adults read more slowly than young adults by making more and longer fixations and more regressions (backward eye movements; e.g., Kliegl, Grabner, Rolfs, & Engbert, 2004; McGowan, White, Jordan, & Paterson, 2014; Paterson et al., 2013; Rayner, Reichle, Stroud, Williams, & Pollatsek, 2006; Rayner, Yang, Schuett, & Slattery, 2013; Stine-Morrow et al., 2010; Whitford & Titone, 2017). Paterson et al. (2013) used word length to investigate if this age-related reading difficulty was due to poorer eye guidance in older age. Word length influenced the probability and duration of eye-fixations on words similarly for young and older adults. Moreover, the two age groups produced similar patterns of landing positions on short and long words (see also Rayner et al., 2006). The findings therefore suggest eye guidance during reading is preserved in older age, at least for alphabetic languages like English.

However, the situation might be different for other writing systems. For many alphabetic languages, eye guidance is aided by the presence of spaces between words, which demarcate word boundaries and provide cues to the length of upcoming words (Rayner, Fischer, & Pollatsek, 1998). Several studies show reading performance suffers more for older adults when these spaces are removed (McGowan et al., 2014; Rayner et al., 2013), suggesting older readers rely particularly heavily on these visual cues to word boundaries. Not all writing systems use spaces to demarcate word boundaries, however. For instance, Chinese is written as a sequence of equally spaced, box-like symbols called characters, some of which correspond to a word although most words in Chinese contain two or more characters (see Li, Zang, Liversedge, & Pollatsek, 2015; Zang, Liversedge, Bai, & Yan, 2011). According to the Lexicon of Common Words in Contemporary Chinese Research Team (2008), only 6% are one-character words, 72% are two characters, 12% three characters, and the remainder mostly four characters. It will therefore be important to establish if there are age differences in eye guidance when reading this unspaced, character-based language.

Existing research aimed at understanding mechanisms of eye guidance during Chinese reading has focused on young adults’ reading behavior and investigated if word length plays as important a role in determining when and where the eyes move as for alphabetic languages (Li et al., 2015; Zang et al., 2011). This research therefore provides a basis for investigating aging effects on eye guidance for this language. The findings for young adults show that long words are skipped less often and fixated for longer than short words (Li, Liu, & Rayner, 2011). However, effects on saccade landing positions differ for words that receive only one first-pass fixation (i.e., fixated once prior to a saccade to another word) or multiple first-pass fixations (Li et al., 2011; Yan, Kliegl, Richter, Nuthmann, & Shu, 2010). Landing positions on words that receive only one first-pass fixation tend to be close to word center for both short and long words, and so differ depending on word length. By contrast, initial fixations on words that receive multiple first-pass fixations tend to land on the first character of words regardless of word length.

Two alternative accounts of these effects have been proposed. According to Yan et al. (2010), the effects show readers select either the beginning or center of words as saccade targets depending on whether they can obtain parafoveal cues to word length. By contrast, Li et al. (2011) argue that the effects are not attributable to parafoveal processing of word length but occur simply because word recognition is facilitated, and the probability of a refixation reduced, when saccades happen to land at an optimal intraword location (i.e., word center). Li et al. also propose that parafoveal processing in Chinese is character- rather than word-based, and that readers achieve processing efficiency by estimating how many upcoming characters they can identify on each fixation and targeting their next saccade to the right of these characters (see also Liu, Reichle, & Li, 2015; Wei, Li, & Pollatsek, 2013). Crucially, however, while the underlying mechanisms differ, both accounts highlight the importance of parafoveal processing for eye guidance during Chinese reading.

Growing evidence indicates that older adults experience age-related reading difficulty for Chinese, which they read more slowly by making more and longer fixations and more regressions than young adults, while also skipping words less frequently and making shorter forward saccades (Wang et al., in press; Zang et al., 2016). These shorter saccades suggest that Chinese older readers might have specific problems with eye guidance (although the age difference in saccade length is small, about 1/3 of a character, and so this requires further investigation). One possibility is that older readers have particular difficulty segmenting unspaced characters into words, possibly because their parafoveal processing of upcoming characters is impaired due to visual decline in older age (see, e.g., Owsley, 2011). Crucially, this may cause older readers to make generally shorter forward saccades which may more rarely land at optimal locations in longer words in particular, and this may be an important source of the reading difficulty they experience.

Accordingly, to investigate age differences in eye guidance during Chinese reading more closely, we recorded the eye movements of young and older adults who read sentences that contained short (2-character) or long (4-character) target words matched for lexical frequency, first-character frequency and predictability. We expected to replicate previously reported aging and word length effects. However, a crucial concern was whether word length effects on fixation times for words would differ across age groups, as this might reveal an important age difference in the processing of words. We also followed the same approach as previous research to assess word length effects on saccade landing positions (Li et al., 2011; Yan et al., 2010) by first examining landing positions overall then specific effects for words that receive only one or multiple first-pass fixations separately. Crucially, these analyses will establish if there are age differences in the likelihood of initially fixating optimal intraword locations, and whether both age groups initially fixate the beginning letters of words that receive multiple fixations, as both factors might affect the efficiency with which words are recognized. We also report additional analyses that examine refixation probabilities as a function of initial landing positions in words and word length effects on the size of forward-moving saccades, to more fully understand how words that receive multiple first-pass fixations are processed. Taken together, the findings will help establish if age differences in eye guidance during Chinese reading make an important contribution to age-related reading difficulty.

## Method

The research was approved by the research ethics committee in the Academy of Psychology and Behavior at Tianjin Normal University and conducted in accordance with the principles of the Declaration of Helsinki.

### Participants

Participants were 26 young adults aged 18–22 years (*M* = 19 years) from Tianjin Normal University, China, and 26 older adults aged 65–89 years (*M* = 77 years) from a residential home for older people in Tianjin. All were native Mandarin speakers, screened for normal acuity (>20/40 in Snellen values) using a Tumbling E eye chart (Taylor, 1978), and nonimpaired cognition using the Beijing version of the Montreal Cognitive Assessment (Nasreddine et al., 2005). Acuity was lower for older adults (*M* = 20/30, range = 20/21 to 20/36) than younger adults (*M* = 20/17, range = 20/12 to 20/21; *t* (50) = 15.04, *p* < .001), as is typical. The two groups were closely matched on years of formal education (young adults, *M* = 13.2 years, range = 13–14 years; older adults, *M* = 13.5 years, range = 9–19 years; *t* < 1) and all participants reported reading for several hours (at least) each week. Vocabulary and short-term memory (STM) were assessed using the Vocabulary Knowledge Test from the Chinese version of the WAIS-III (Wechsler, Chen, & Chen, 2002) and the WAIS-III digit-span subtest (Wechsler, 1997). Vocabulary scores were similar for young (*M* = 69.9, *SD* = 5.6) and older adults (*M* = 70.3, *SD* = 5.8; *t* < 1), and digit spans lower for older (*M* = 12.4, *SD* = 2.6) than younger adults (*M* = 15.0, *SD* = 2.0; *t* (50) = 4.07, *p* < .001), as is typical (Ryan, Sattler, & Lopez, 2000).

### Stimuli and Design

**Figure 1 fig1:**
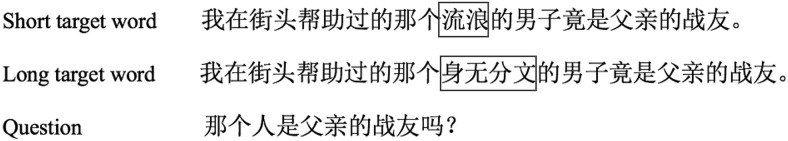
Example sentence and comprehension question. Example sentence containing a short and long word and the accompanying comprehension question. The sentences translate as “The homeless/penniless man I helped on the street was my father’s comrade-in-arms.” The question translates as “Was the man my father’s comrade-in-arms?” Target words are shown in boxes but were presented normally in the experiment.

Stimuli were 64 sentence frames that contained a short (2-character) or long (4-character) target word (see Figure 1 & Appendix). The short and long words were closely matched for log lexical frequency (long words, *M* = 1.94, short words, *M* = 1.99; *t* < 1) and log first-character frequency (short words, *M* = 3.9, long words, *M* = 4.3; *t* < 1.7) using the SUBTLEX-CH corpus (Cai & Brysbaert, 2010). The 2-character words could not form a word with an adjacent character in the sentences, and the first 2 characters of the 4-character words could not form a word. Naturalness ratings from 16 readers who did not participate in the experiment showed that sentences were highly natural (*M* = 6.4, max = 7), with no difference due to word length (short words, *M* = 6.3, long words, *M* = 6.4; *t* < 1). A cloze task with another 11 readers showed that short and long target words were equally unpredictable in the sentence frames (short words, *M* = 1.5%, and long words, *M* = 0.7%, words guessed correctly; *t* < 1.2). A recognition test administered after the experiment confirmed all participants knew the meanings of all the target words. The sentences were 16–28 characters (*M* = 24) long and target words were always located near the middle of sentences.[Fig-anchor fig1]

Sentence frame and target word combinations were divided into two lists, each containing all 64 frames and equal numbers of short and long target words. Thirteen participants from each age group were randomly allocated to each list. The design was therefore mixed, with the between-participants factor age group (young adult, older adult) and within-participants factor word length (long, short). Sentences in each list were presented in random order, preceded by four practice sentences.

### Apparatus and Procedure

An EyeLink 1000 eye-tracker recorded each participant’s right-eye gaze location every millisecond during binocular viewing. Stimuli were presented in Song font as black text on a white background. Each character subtended 0.9° approximately and so was of normal size for reading. Participants took part individually. At the start of the experiment, each participant was instructed to read normally and for comprehension, and a 3-point horizontal calibration procedure ensured spatial accuracy <.35°. Thereafter, calibration accuracy was checked before each trial and the eye-tracker recalibrated as necessary. At the start of each trial, a fixation square equal in size to one character was presented on the left side of the screen. Once this was fixated, a sentence was presented with the first character replacing the square. The participant pressed a response key once they finished reading each sentence. The sentence then disappeared and was replaced on 25% of trials by a yes/no comprehension question, to which the participant responded by pressing a response key. The experiment lasted 45 min for each participant.

## Results

Following standard procedures, fixations less than 80ms and greater than 1200ms were removed. Trials also were excluded if track-loss or error occurred (affecting <1% of trials). Data were analyzed using the lme4 package (Bates, Mächler, Bolker, & Walker, 2014) in R (R Development Core Team, 2016). Linear mixed-effects models were used for continuous variables and generalized linear models for dichotomous variables. Maximal random effects were used for both types of model (Barr, Levy, Scheepers, & Tily, 2013). The pattern of effects did not differ between log-transformed and untransformed data, so analyses of untransformed data are reported for transparency. For all analyses, *t/z* > 1.96 were considered significant as with high degrees of freedom (as in our analyses) *t* > 1.96 produces *p* values where *p* < .05. Participants and stimuli (sentences in sentence-level analyses and target words in word-level analyses) were specified as crossed-random effects. Age-group was a fixed factor in sentence-level models, and age-group and word length were fixed factors in word-level models. Response accuracy for comprehension questions was >80% for all participants (*M* = 96%) and did not differ across age groups (*t* < 1.6). See Table 1 and 2 for sentence-level and target word means and Tables 3 and 4 for a summary of statistical effects.[Table-anchor tbl1][Table-anchor tbl2][Table-anchor tbl3][Table-anchor tbl4]

### Sentence-Level Analyses

Compared to young adults, the older adults read more slowly, and made more and longer fixations and more regressions, consistent with age-related reading difficulty. Compared to young adults, the older adults also made shorter forward saccades and skipped target words infrequently, consistent with findings in other recent Chinese studies.

### Word-Level Analyses

Compared to young adults, the older adults skipped words less frequently, and had longer first-fixation durations, higher refixation probabilities, and longer gaze durations and total reading times for target words, consistent with age-related reading difficulty. We also observed clear word length effects, due to lower word-skipping, higher refixation probabilities and longer gaze durations and total reading times for long compared to short words. Crucially, word length effects for gaze durations and total reading times were qualified by interactions with age group, due to larger effects of word length for the older than younger adults. First-fixations were shorter for the long than short words for both age groups. This was most likely because long words received more refixations than short words, and so effects of word length on fixation times were observed clearly only in fixation time measures that include all the fixations made during the initial or overall processing of words (i.e., gaze duration and total reading time, respectively).

### Word-Level Landing Position Effects

Mean landing positions were closer to word beginnings for the older than younger adults and for the long than short words. The launch sites of saccades that ended in these fixations were nearer the beginning of target words (and so saccades were shorter) for the older than younger adults and for the short than long words. The indication, therefore, is that older readers made shorter saccades that landed nearer the beginnings of words. We explored these effects further by analyzing landing positions separately for words that received only one or multiple first-pass fixations, following Yan et al. (2010) and Li et al. (2011). The percentage of trials in which words received one first-pass fixation (i.e., the inverse of refixation probability) was greater for the young adults (short words, young adults = 85%, older adults = 60%; long words, young adults = 50%, older adults = 15%), suggesting they recognized words more efficiently.

### Landing Positions on Words Receiving One First-Pass Fixation

**Figure 2 fig2:**
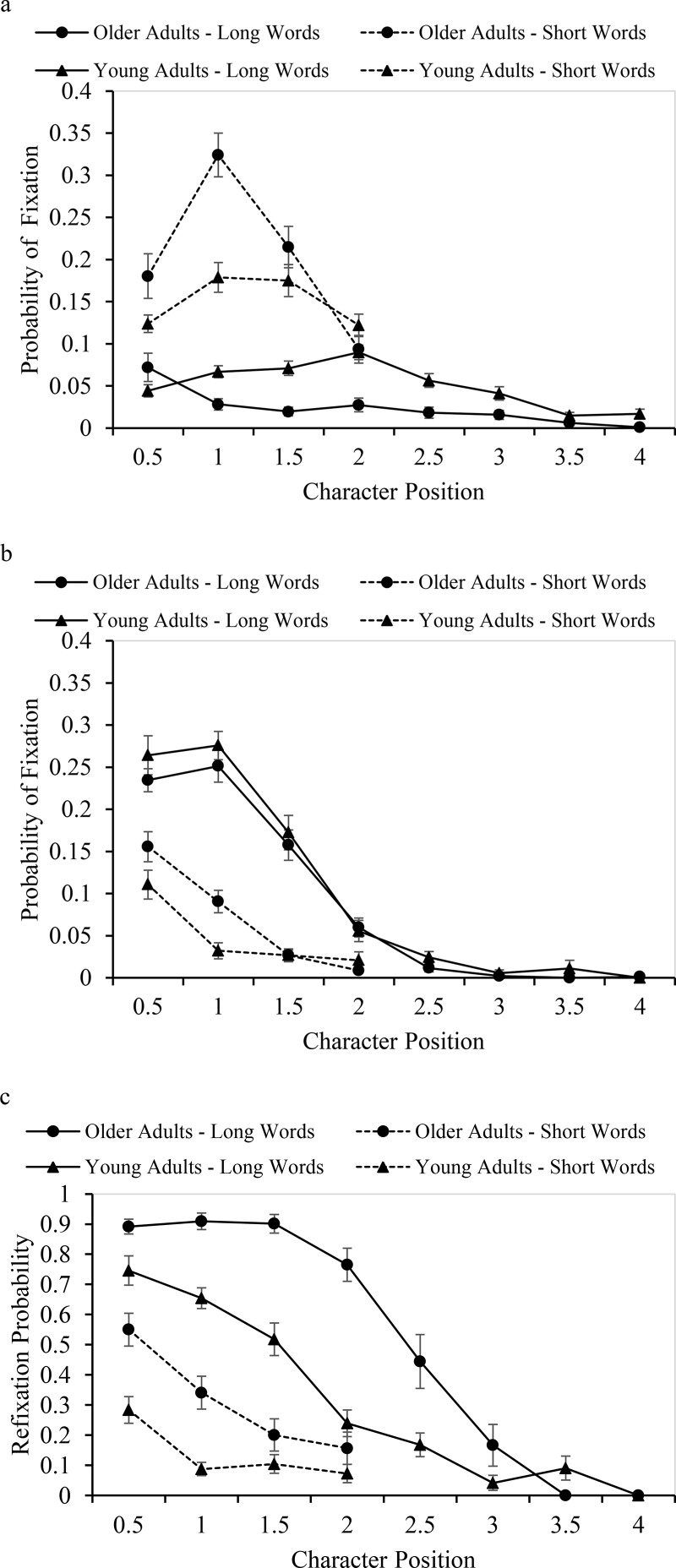
Landing positions of initial fixations in words receiving (a) one first-pass fixation or (b) multiple first-pass fixations, and (c) re-fixation probability as a function of initial landing position. Note that differences in the amplitude of curves for young and older adults reflect overall differences in word-skipping, single-fixation, and refixation probabilities for the young and older adults.

Landing positions for words receiving one first-pass fixation were closer to the beginning of words for the older than younger adults and for the long than short words but with no interaction between age group and word length. Figure 2a shows the proportion of fixations at each half-character position. The distributions, for the young adults in particular, appear to peak near the center of short and long words.[Fig-anchor fig2]

### Initial Landing Positions on Words Receiving Multiple First-Pass Fixations

Landing positions for words receiving multiple first-pass fixations were closer to the beginning of long than short words but did not vary with age group. Figure 2b shows the proportion of fixations at each half-character position. Saccades tended to land near the beginning of words for both age groups, resembling previous findings for young adults (Li et al., 2011; Yan et al., 2010). Taken together, the findings show a higher likelihood of readers initially fixating an optimal location in words that receive only one rather than multiple first-pass fixations. Moreover, as the older readers made fewer single-fixations on words (and especially longer words) than young adults, it appears they are less likely to initially fixate a word at an optimal location (i.e., word center).

### Re-Fixation Probability as a Function of Initial Landing Position

In alphabetic languages, refixation probabilities are higher when saccades land at the beginning or end rather than middle of words (Nuthmann, Engbert, & Kliegl, 2005; Rayner et al., 1996). Such effects are often attributed to mis-located fixations, due to saccades undershooting or overshooting word boundaries. Figure 2c shows refixation probabilities for landing positions at different intraword locations in the present experiment. These were analyzed by comparing refixation probabilities following initial fixations on the first and second characters of short words, and first two half-characters, middle two half-characters, and end two half-characters of long words. For the short words, refixation probabilities were higher following initial fixations on the first than second characters (β = .20, *SE* = .04, *t* = 5.28) with no interaction with age group (β = .18, *SE* = .04, *t* < 2). For the long words, refixation probabilities were higher following initial fixations at beginning rather than middle locations (β = .33, *SE* = .03, *t* = 9.45), and middle rather than end locations (β = .42, *SE* = .05, *t* = 5.40), with a similar pattern for young and older adults. The pattern replicates that reported previously for young adults (Li et al. (2011). Crucially, the findings suggest landing position effects on words receiving multiple fixations in the present experiment are not due to mis-located fixations (as saccades rarely overshot word boundaries) but consistent with readers sequentially processing successive portions of words.

### Outgoing Forward Saccades from Target Word

Outgoing forward saccades are longer from long than short words if processing is word-based (Wei et al., 2013). In the present experiment, there was an interaction between age group and word length, which we examined by comparing the size of the word length effect for the young and older adults. This revealed that the interaction was due to a larger word length effect for young than older adults (β = .35, *SE* = .07, *t* = 4.86), and so suggests that the older adults were less likely to process words using a word-based strategy.

## Discussion

Our results confirm that Chinese older adults read more slowly than young adults by making more and longer fixations, more regressions, shorter forward saccades, and skipping word infrequently (Wang et al., in press; Zang et al., 2016). The results also reveal potentially important age differences in the effects of word length. For both age groups, long words were skipped less often and fixated for longer than short words. In these respects, the findings accord with those for alphabetic languages (Brysbaert et al., 2005; Joseph et al., 2009; Paterson et al., 2013, 2015; Rayner, 1979; Rayner & McConkie, 1976; Rayner et al., 1996). But, unlike previous studies that examined aging effects in alphabetic languages (Paterson et al., 2013), word length effects were larger for older than younger adults, due to older readers making disproportionately longer fixations on longer words. These findings are theoretically important because they reveal age differences in word length effects for unspaced, character-based languages like Chinese which have not been observed for spaced, alphabetic languages like English. The findings may also have practical implications for understanding aging effects on Chinese reading, as they suggest Chinese older readers have particular difficulty recognizing long words.

Analyses that examined saccade landing positions on target words in sentences shed further light on this age difference in word length effects. We followed an established approach and examined landing positions separately for words that received only one or multiple first-pass fixations (Li et al., 2011; Yan et al., 2010). For the young adults, we replicated findings showing saccades to words that receive only one first-pass fixation tend to land near word center, whereas saccades to words that receive multiple first-pass fixations are more likely to land at the beginning of words. These effects are attributed to flexible targeting of saccades toward the beginning or center of words depending on the availability of parafoveal word length cues (Yan et al., 2010), or the reduced likelihood of a refixation if a saccade just happens to land at an optimal intraword location (i.e., word center) that facilitates word recognition (Li et al., 2011). Our findings show that Chinese young and older adult readers produce very similar patterns of landing positions, so that both tend to initially fixate the beginning of words that receive multiple fixations and fixate near the center of words that receive only one fixation. However, as the older adults more rarely make single-fixations on words, it also seems clear that they benefit less from fixations that land at optimal locations in words.

This pattern of effects may be due to older readers making generally shorter forward saccades, possibly due to impaired eye guidance. This may reflect specific difficulties segmenting text and assembling characters into words or be a consequence of poorer parafoveal processing due to visual declines in older age (see, e.g., Owsley, 2011). In particular, if Chinese older readers have difficulty processing characters parafoveally, they may make generally shorter saccades than young readers because they have difficulty identifying word boundaries (e.g., Yan et al., 2010) or can recognize fewer upcoming characters on each fixation (Li et al., 2011; Liu et al., 2015; Wei et al., 2013). In either case, this may lead older readers to more rarely make saccades that land at optimal intraword locations, for long words in particular, which may help explain the difficulty they experience.

A further possibility is that word recognition difficulty leads readers to use a character- rather than word-based strategy to process words that receive multiple fixations. Evidence for this comes from the finding that refixation probabilities decreased linearly following initial fixations at beginning, middle or end locations in long words. This contrasts with findings from alphabetic languages showing readers are more likely to make a corrective refixation when their initial fixation lands at a suboptimal location (i.e., toward the beginning or end of words rather than word center; e.g., Nuthmann et al., 2005; Rayner et al., 1996). It suggests the processing of Chinese words that receive multiple first-pass fixations may not be similarly word-based (see Supplementary Materials for further evidence). Moreover, as older readers more often make multiple fixations on words, they may be less likely than young adults to use a word-based reading strategy. Readers typically make longer forward-moving saccades from long than short words if processing is word-based (Wei et al., 2013). Accordingly, our finding that word length effects on saccade length is greater for young than older adults accords with this possibility and provides further evidence that older adults are more likely to use a character-based strategy to recognize words. However, this possible age difference in Chinese reading strategy will require further investigation. In particular, whether it reflects poorer parafoveal processing or word-segmentation processes due to sensory and cognitive declines is unclear. Further research on the underlying mechanisms is therefore essential to better understand the difficulties experienced by older readers of unspaced, character-based languages like Chinese.

## Supplementary Material

10.1037/pag0000258.supp

## Figures and Tables

**Table 1 tbl1:** Means for Sentence-Level Measures

	Age-Group	
Measure	Young Adult	Older Adult
Sentence reading time (ms)	4260 (43)	8033 (89)
Average fixation duration (ms)	235 (1)	281 (1)
Number of fixations	15.4 (.15)	24.8 (.25)
Forward saccade length (characters)	2.9 (.02)	2.1 (.02)
Number of regressions	4.1 (.07)	6.0 (.10)
*Note*. Sentence reading time is the time from the onset of the sentence presentation until the participant presses a key to indicate they have finished reading. Number of fixations is the count of fixations made during sentence reading. Average fixation duration is the mean duration, in milliseconds, of these fixations. Forward saccade length is the mean length, in characters, of progressive eye movements. For each measure, the standard error of the mean is shown in parentheses.		

**Table 2 tbl2:** Means for Target Word-Level Measures

	Age-Group			
	Young Adult		Older Adult	
Measure	Short	Long	Short	Long
Word-skipping (%)	17 (1)	3 (1)	4 (1)	.5 (.2)
First-fixation duration (ms)	251 (3)	237 (3)	321 (4)	313 (4)
Re-fixation probability	.15 (.01)	.5 (.02)	.40 (.02)	.85 (.01)
Gaze duration (ms)	286 (5)	376 (7)	488 (12)	811 (18)
Total reading time (ms)	404 (9)	556 (12)	701 (19)	1117 (24)
Initial landing position (all trials)	48 (1)	31 (1)	28 (1)	22 (1)
Launch site (characters)	1.70 (.05)	1.78 (.05)	1.08 (.03)	1.25 (.03)
Landing position (one first-pass fixation)	50 (1)	41 (1)	46 (1)	35 (2)
Landing position (multiple first-pass fixations)	31 (3)	21 (1)	26 (1)	19 (1)
Outgoing forward saccade length (characters)	3.22 (.05)	3.94 (.06)	2.32 (.03)	2.66 (.04)
*Note*. Word-skipping is the probability of not fixating a word during first-pass reading (prior to a fixation to the word’s right). First-fixation duration is the length of the first first-pass fixation on a word. Re-fixation probability is the probability a word receives more than one first-pass fixation. Gaze duration sums all first-pass fixations on a word (prior to a saccade to the right of the word or a regression to the left). Total reading time sums all the fixations on a word. Landing position is the percentage distance in from a word’s left boundary to the first first-pass fixation on that word, reported for all words and for words receiving only one or multiple first-pass fixations. Launch site is the distance, in characters, backwards from a word’s left boundary to the starting point of the saccade that terminates in the first first-pass fixation on the word. Outgoing forward saccade length is the length, in characters, of forward moving saccades away from a word. For all measures, the standard error of the mean is shown in parentheses.				

**Table 3 tbl3:** Statistic Effects of Age-Group for Sentence-Level Analyses

Measure	β	*SE*	*t*
Sentence reading time	3785.5	589.4	6.42***
Average fixation duration	46.74	8.94	5.23***
Forward saccade amplitude	.79	.17	4.54***
Number of fixations	9.43	1.67	5.66***
Number of regressions	1.95	.64	3.06**
** *p* < .01. *** *p* < .001.			

**Table 4 tbl4:** Statistical Effects of Age-Group, Word Length, and Age-Group × Word Length for Word-Level Analyses

Measure	Effect	β	*SE*	*t/z*
Skipping rate	Age-Group	1.81	.45	4.05***
	Word Length	2.16	.29	7.33***
	Age-Group × Word Length	.02	.59	.03
First-Fixation duration	Age-Group	74.53	11.43	6.52***
	Word Length	9.78	3.46	2.82**
	Age-Group × Word Length	6.12	6.93	.88
Re-Fixation probability	Age-Group	1.80	.26	6.93***
	Word Length	2.34	.14	17.14***
	Age-Group × Word Length	.49	.27	1.83^†^
Gaze duration	Age-Group	321.05	54.98	5.84***
	Word Length	212.34	9.82	21.63***
	Age-Group × Word Length	235.52	19.63	12.00***
Total reading time	Age-Group	433.55	76.45	5.67***
	Word Length	290.40	13.99	20.76***
	Age-Group × Word Length	269.21	51.92	5.19***
Landing position	Age-Group	.10	.02	4.82***
	Word Length	.17	.01	22.01***
	Age-Group × Word Length	.00	.02	.28
Launch site	Age-Group	.62	.15	4.02***
	Word Length	.10	.04	2.32*
	Age-Group × Word Length	.11	.09	1.21
Landing position (one first-pass fixation)	Age-Group	.06	.02	2.82**
	Word Length	.11	.01	7.71***
	Age-Group × Word Length	.02	.03	.75
Initial landing position (multiple first-pass fixations)	Age-Group	.03	.02	1.34
	Word Length	.09	.02	4.19***
	Age-Group × Word Length	.04	.05	.91
Outgoing forward saccade length (characters)	Age-Group	1.18	.20	6.03***
	Word Length	.50	.05	9.48***
	Age-Group × Word Length	.33	.10	3.30**
^†^ = *p* < .1. * = *p* < .05. ** = *p* < .01. *** = *p* < .001.
