# Supplementary material for: Effects of Word Length on Eye Guidance Differ for Young and Older Chinese Readers
Source: Psychol Aging. 2018 Jun;33(4):685–92. doi: 10.1037/pag0000258 (PMC6001944; doi:10.1037/pag0000258)
Supplement: Supplementary file 1 [file z2m-3141_PAG-2017-1284_SUPPL.zip › z2m999183141so1.docx]

**Supplemental Materials**

**Effects of Word Length on Eye Guidance Differ for Young and Older Chinese Readers**

**By S. Li et al., 2018, *Psychology and Aging***

**http://dx.doi.org/10.1037/pag0000258**

As supplementary materials, we include figures that illustrate the location of re-fixations on target words that received multiple first-pass fixations and a list of sentence stimuli with English translations.

Re-Fixation Locations on Target Words.

Following an approach taken in studies of reading development (Joseph, Liversedge, Blythe, White, & Rayner, 2009; McConkie, Zola, Grimes, Kerr, Bryant & Wolff, 1991; Zang, Liang, Bai, Yan, & Liversedge, 2013), we conducted exploratory analyses that examined the locations of re-fixations on words receiving multiple first-pass fixations. As noted in the results section of the paper, the young adults made at least one re-fixation on 15% of short words and 50% of long words, and the older adults made at least one rerefixation on 40% of short words and 85% of long words. Consequently, while words frequently received multiple first-pass fixations, there is clearly an imbalance in the number of trials that contribute to effects for each age group and words of each length. Moreover, it seems likely that these trials will not be well-balanced for important word characteristics (e.g., word frequency, character complexity). Consequently, while we considered that an analysis of the location of re-fixations might be helpful in furthering our understanding of the nature of the reading strategy used by young and older adults when words receive multiple first-pass fixations, we must also acknowledge that because these are naturalistically occurring data, the results should be treated with caution.

A first analysis examined the location of second fixations on the target words. It was not possible to conduct formal analyses on these data. However, an inspection of Figure 1 shows that young adults’ second fixations mostly landed in the second half of words. By contrast, older adults’ second fixations mostly landed in the second half of short words and near the center of long words. The data for first-fixation landing positions reported in the paper (and in previous studies) show that readers tend to make initial fixations that land near the beginning characters in words that receive multiple first-pass fixations. The second-fixation data we report here are therefore consistent with readers using a left-to-right serial processing strategy according to which they sequentially fixate portions of multiple-character words. For the young adults, this appears to involve fixating each half of a word, whereas the older adults appear to process longer words by fixating the beginning character and then middle portion of words with their first two fixations.

Figure 1. *Locations of Second Fixations in Target Words Receiving Multiple First-Pass Fixations (Error Bars Show the Standard Error of the Mean)*

To explore this effect of re-fixation location further, we also examined the location of third fixations on the target words. The young adults made too few third fixations to merit examining the locations of these fixations (only 1% for short words and 10% for long words). Older adults also made relatively few third fixations on short words (only 10%) and, as Figure 2 shows, the locations of these fixations did not vary systematically. However, older adults made more third fixations on long words (47% of words) and, as Figure 2 illustrates, the locations of these fixations peaked towards the end character in words (Figure 2d). This provides a further indication that readers linearly inspect the characters in words serially from left-to-right and that older readers do this for longer words more slowly and with more fixations than young adults. Taken together, the pattern of re-fixation locations is consistent with young and older adult readers using a character- rather than word-based strategy to recognize word that receive multiple first-pass fixations. Moreover, as older readers are more likely to make multiple first-pass fixations on words, especially when these are longer, it appears they may also be more likely to use a character-based reading strategy to recognize words, and this may be an important component of their much slower reading behavior.

Figure 2. *Locations of Third Fixations in Target Words Receiving Multiple First-Pass Fixations (Error Bars Show the Standard Error of the Mean)*

Sentence Stimuli

The sentence frames and target words used in the experiment are listed below. The short or long target words are shown in square brackets separated by a slash (‘\’). Translations into English are shown below each sentence.

1.我在街头帮助过的那个 [流浪/身无分文] 的男子竟是父亲的战友。

The homeless / penniless man I helped on the street was my father’s comrade-in-arms.

2.新政权开明的文教政策得到了广大 [学者/知识分子] 的支持和拥护。

The enlightened culture and education policy of the new regime has won the support of the broad mass of [scholars / intellectuals].

3. 他在工作中表现出的 [踏实/实事求是] 的态度赢得了大家的认可。

His [down-to-earth / realistic] attitude to work helps him win everyone's recognition.

4. 邻居家赵大爷因 [煤气/一氧化碳] 中毒抢救无效不幸去世。

Mr. Zhao, who lived next door, died because of [gas / carbon monoxide] poisoning

5. 侦探的工作就是要从 [杂乱/错综复杂] 的线索中分析出案情的主要脉络。

Seeing the whole picture of a case from [unorganized / complex] cues is the main job of a detective.

6. 场下许多观众都 [不由/情不自禁] 地为剧中主人公的悲惨遭遇而落泪。

Many of the audience [couldn’t help / couldn’t help] weeping for the protagonist’s tragic plight.

NB. 不由 and 情不自禁 have the same meaning in this context.

7. 爆破专家正在 [谨慎/小心翼翼] 地排查可能安放炸药的体育馆。

Explosives experts are investigating very [cautiously / carefully] at a gym where a bomb might have been installed.

8. 手持站票的旅客们 [拼命/争先恐后] 地往前挤以便占据一个有利的位置。

Passengers who were holding standing tickets mobbed forward [like mad / falling over each other] in order to get a good position.

9. 大赛组委会的这种安排 [无疑/毫无疑问] 是为了平衡各大洲利益。

[No doubt / Undoubtedly] the organizing committee made this arrangement in order to balance the benefits for competitors from all continents.

10. 文学院张启明教授 [独特/与众不同] 的授课风格给大家留下了深刻印象。

Zhang Qiming, who is a professor at the school of literature, impressed everyone by his [unique / unusual] teaching style.

11. 徐经理的办公桌上 [杂乱/乱七八糟] 地堆放着各部门送来的几十份文件。

Dozens of files from different departments are [cluttered / in a jumble] on manager Xu’s desk.

12. 我就算沦落到 [行乞/无家可归] 的地步也决不低头接受你的施舍。

Even if I end up [a beggar / homeless], I would never accept your help.

13. 这款游戏允许用户 [任意/随心所欲] 地调整城市的地形和植被。

Users can adjust the terrain and vegetative cover of the city in the game [discretionarily / at will].

14. 医生建议小王先做一个 [切片/核磁共振] 检查以排除最坏的可能性。

The doctor suggests Xiao Wong has [a biopsy / an MRI exam] to rule out the worst case.

15. 你说的那个毕业后整天 [闲逛/无所事事] 的小伙子就是老刘家的孩子。

The young man with whom you talked, who [wanders around / does nothing] every day after graduation, is Lao Liu’s son.

16. 老李纵容子女 [胡闹/为所欲为] 的态度是极为不负责任的。

It is completely irresponsible of Lao Li to tolerate his children [running wild / doing whatever they like].

17. 曾经跑完五公里就 [晕倒/精疲力尽] 的我根本没有能力尝试马拉松。

I’m not able to run marathon because I [fainted / collapsed] once after a only 5-kilometre race.

18. 无论结果怎样我们都会 [坚决/一如既往] 支持项目组的决策。

We will support the project team’s decision [firmly / as always] whatever the outcome.

19. 他趁大伙没注意的时候 [悄悄/鬼鬼祟祟] 地溜进了档案室。

He slipped into the archives [quietly / furtively] while everyone was paying no attention.

20. 刘助理议在容器中填充 [氮气/二氧化碳] 以防止实验材料因过热而自燃。

Assistant Liu suggests pumping some [nitrogen / carbon dioxide] into the device to prevent it from self-igniting due to overheating.

21. 我们出席会议的目的正是要 [坦诚/开诚布公] 地与大家交流讨论。

We attend this meeting because we want to speak with each other [frankly / sincerely and openly].

22. 犯罪嫌疑人的 [行踪/一举一动] 完全处于专案组干警的监视之下。

The suspect’s [whereabouts / every movements] is under the surveillance of the police.

23. 不考察实际情况就盲目 [指挥/发号施令] 的行为很有可能埋下隐患。

[Commanding others / Issuing orders] without knowing the specific conditions is potentially hazardous.

24. 李栋的祖父年轻时是一位 [杰出/才华横溢] 的音乐剧演员。

When Li Dong’s grandfather was young, he was [an outstanding / a talented] stage musical performer.

25. 河对岸的敌军 [试探/虚张声势] 了大半天也没敢真正发动进攻。

The enemy on the other side of the river didn’t begin to attack for real but [tested our situation/ bluffed] for a while.

26. 书中把小张写成一个 [厌世/嫉恶如仇] 的青年学生。

The book describes Xiao Zhang who as a young student [was a misanthrope / despised evil deeds]

27. 闫志南从小就立志要成为 [牛顿/爱因斯坦] 那样伟大的物理学家。

Since Yan Zhinan was a little boy, he was determined to be a great physicist like [Newton / Einstein].

28. 正在晨练的王大爷 [果断/毫不犹豫] 跳入冰冷的湖中搭救落水儿童。

Mr. Wang, who happened to be doing morning exercises nearby, jumped into the cold water [decisively / without hesitation] and saved the drowning child.

29. 登山队全体成员都 [平安/安然无恙] 地返回了山脚下的营地。

All the climbers returned to the campsite at the foot of the hill [safely / safe and sound].

30. 整天买彩票幻想幸运 [光顾/从天而降] 还不如踏踏实实好好工作。

You should take your job seriously rather than hoping luck [arrives / is on your side] so you win a lottery.

31. 狠心的工头看着大家累到 [虚脱/筋疲力尽] 也不允许停工休息。

Although the workers were all [in a state of collapse / exhausted], the foreman refused to let them take a break.

32. 礼堂地下的防空洞里挤满了 [受惊/惊慌失措] 的老人和小孩。

The bomb shelter hidden beneath the hall was crowded with [frightened / panic] children and elderly people.

33. 被凶猛的猎豹逼到 [绝境/走投无路] 的羚羊正做着最后的挣扎。

The antelope, driven into [despair / a corner] by a ferocious cheetah, is putting up a last-ditch fight.

34. 对那天发生的事情一直 [回避/守口如瓶] 的老刘引起了大家的怀疑。

Lao Liu [always avoids talking / won’t ever say a word] about what happened the other day, which arouses our suspicions.

35. 过去整天在学校 [闯祸/游手好闲] 的小王竟然创业开了一家修车行。

Xiao Wong, who used to [get into trouble / laze about] at school, opened a garage by himself.

36. 确保九江市内各处堤防 [稳固/万无一失] 是此次抗洪的核心任务。

The key thing to fight against the flood this time is to make sure all the dykes in Jiujiang city are [stable / 100 percent foolproof].

37. 假期结束后我们要以 [崭新/焕然一新] 的精神面貌迎接新的学期。

After the vacation, we should greet the new semester with [a new mental outlook / fresh sprit].

38. 朝韩两国关系 [和睦/重归于好] 必将促进东北亚地区的和平与繁荣。

South and North Korean [remaining peaceful / staying in concord] with each other will certainly contribute to the peace and prosperity of Northeast Asia.

39. 此类充斥迷信色彩的 [谣言/无稽之谈] 正是邪教组织迷惑群众的诡计。

This kind of superstitious [rumour / nonsense words] is exactly what religious cults want to spread to deceive the masses.

40. 警方根据犯罪现场遗留的 [脚印/蛛丝马迹] 成功锁定了嫌疑人。

The police successfully identified the suspect from the [footprint / clues] left at the crime scene.

41. 因为这点小事而 [纠结/耿耿于怀] 好几个星期实在是没必要。

It’s not necessary to be [bothered / rankled] by such a trifle for weeks.

42. 即便你把谎言编到 [极致/天衣无缝] 也休想瞒过所有的人。

You can never fool everyone even if your lie is [perfect / flawless].

43. 外行们自以为 [严谨/无懈可击] 的逻辑在专家看来漏洞百出。

The logic which seems to be [rigorous / impeccable] to the layman is actually full of contradictions to the professional.

44. 这一计划是董事会各位成员 [权衡/深思熟虑] 之后的最优方案。

After the directorate’s [balancing / careful consideration], this scheme is believed to be the best.

45. 他那副一见别人落难就 [得意/幸灾乐祸] 的样子令人不齿。

It is a complete disgrace that he [is satisfied by / takes pleasure in] other people’s misfortune.

46. 民国三十五年那起投毒案的 [元凶/罪魁祸首] 直到解放后才被绳之以法。

The [culprit / villain] in the poisoning case in the 35th year of the Republic of China wasn’t brought to justice until after the War of Liberation.

47. 能与单反相机性能 [媲美/相提并论] 的拍照手机只存在于广告中。

This kind of mobile phone, which has a camera function that [can compare / is equal] to that of an SLR camera, only exists in advertisements.

48. 他之前那些 [恶劣/忘恩负义] 的行径无疑令养父母伤心不已。

No doubt his [abominable / ungrateful] behaviour has broken his adoptive parents’ hearts.

49. 魔术师仅凭一两个节目就能 [成名/出人头地] 的时代已经是历史了。

The time in which magicians can [make a name for themselves / stand out] due to only one or two programs is in the past.

50. 要想这次演出不 [出丑/重蹈覆辙] 就必须对前两幕的剧情进行修改。

You must have a different plot in the first two seasons if you do not want to [make a fool of yourself / make the same mistake] during the show.

51. 这起事故不查到 [根源/水落石出] 就无法彻底排除安全隐患。

If we cannot [find the cause / get to the bottom] of this accident, we can never eliminate the danger hidden behind it.

52. 像张国栋这样 [卓越/出类拔萃] 的人才是不会长久留在你们公司的。

People like Zhang Guodong, who is so [excellent / outstanding], will never stay with your company for long.

53. 因车辆机械故障而在比赛中 [失利/半途而废] 令车手懊恼不已。

[Losing the racing / Giving up the racing halfway] because of the mechanical fault of the car annoyed the driver so much.

54. 两年前那届规模 [空前/史无前例] 的世界杯必将永载体育史册。

The [unprecedented / unparalleled] world cup two years ago will go down in sports history forever.

55. 殴打安检员并在候车厅 [撒野/无理取闹] 的乘客已被警方逮捕。

The passenger who beat a security inspector and behaved [atrociously / unreasonably] in the waiting room has been arrested by the police.

56. 指望他那种优柔寡断的人给个 [痛快/直截了当] 的回答可真不容易。

It’s so difficult to get a [quick / straight] answer from such an indecisive person.

57. 我不是为了听你这些 [闲话/陈词滥调] 才大老远跑到上海来的。

I’ve not travelled far from Shanghai just to listen to your [chitchat / platitudes].

58. 外国侵略者对 [逃难/手无寸铁] 的平民进行屠杀的行径令人发指。

The invaders’ barbarous act of slaughtering [refugees / unarmed civilians] is totally odious.

59. 西南边边境一些 [偏远/与世隔绝] 的山区直到前几年才修通了公路。

Some [remote / secluded] mountainous regions in the Southwest has had road only in the last few years.

60. 上一任副局长因 [渎职/玩忽职守] 被判入狱三年零六个月。

The last vice-director was sentenced to three years and six months in prison because of [malfeasance / negligence].

61. 改革的第一步是要摒弃那些 [守旧/循规蹈矩] 的做派和思维方式。

The first step of the reform is to abandon [conservative / prim] behaviours and ways of thinking.

62. 他伤愈归来后被安排做一些 [简易/力所能及] 的辅助性工作。

He has been told to do [simple work / work within his ability] after recovering from the injury.

63. 二床的患者直到生命 [垂危/奄奄一息] 之际也没有亲友来探望。

The patient in the second bedroom has no relatives or friends to visit even though he is [dying / at his last gasp].

64. 女排姑娘们这种 [拼搏/勇往直前] 的精神值得我们各行各业学习。

The [tenacious / plucky] spirit of the Chinese women’s volleyball team is an inspiration to people from all walks of life.
